# Kinetically Consistent Data Assimilation for Plant PET Sparse Time Activity Curve Signals

**DOI:** 10.3389/fpls.2022.882382

**Published:** 2022-07-22

**Authors:** Nicola D'Ascenzo, Qingguo Xie, Emanuele Antonecchia, Mariachiara Ciardiello, Giancarlo Pagnani, Michele Pisante

**Affiliations:** ^1^School of Life Science and Technology, Huazhong University of Science and Technology, Wuhan, China; ^2^Department of Medical Physics and Engineering, Istituto Neurologico Mediterraneo, Istituto di Ricovero e Cura a Carattere Scientifico, Pozzilli, Italy; ^3^Department of Electronic Engineering and Information Science, University of Science and Technology of China, Hefei, China; ^4^Faculty of Bioscience and Technology for Food, Agriculture and Environment, University of Teramo, Teramo, Italy

**Keywords:** data-driven digital signal processing for plant imaging, data assimilation algorithms, kinetic modeling, dynamic plant positron emission tomography, functional plant imaging, portable imaging device, plant physiology

## Abstract

Time activity curve (TAC) signal processing in plant positron emission tomography (PET) is a frontier nuclear science technique to bring out the quantitative fluid dynamic (FD) flow parameters of the plant vascular system and generate knowledge on crops and their sustainable management, facing the accelerating global climate change. The sparse space-time sampling of the TAC signal impairs the extraction of the FD variables, which can be determined only as averaged values with existing techniques. A data-driven approach based on a reliable FD model has never been formulated. A novel sparse data assimilation digital signal processing method is proposed, with the unique capability of a direct computation of the dynamic evolution of noise correlations between estimated and measured variables, by taking into explicit account the numerical diffusion due to the sparse sampling. The sequential time-stepping procedure estimates the spatial profile of the velocity, the diffusion coefficient and the compartmental exchange rates along the plant stem from the TAC signals. To illustrate the performance of the method, we report an example of the measurement of transport mechanisms in zucchini sprouts.

## 1. Introduction

The extraction of quantitative plant transport parameters from the sparse time activity curve (TAC) signals measured with positron emission tomography (PET) techniques represents one of the frontiers of plant digital imaging (Hubeau and Steppe, 2015; Galieni et al., 2021; Mincke et al., 2021a; Antonecchia et al., 2022), with a strong impact in early stress assessment (Tsukamoto et al., 2008; Yoshihara et al., 2014; Partelová et al., 2017), yield improvement (Yamazaki et al., 2015; Hubeau et al., 2019b; Mincke et al., 2020a), sustainable agriculture (Karve et al., 2015; Kuritaa et al., 2020) and climate change studies (Hubeau et al., 2019a).

**Figure 1:**
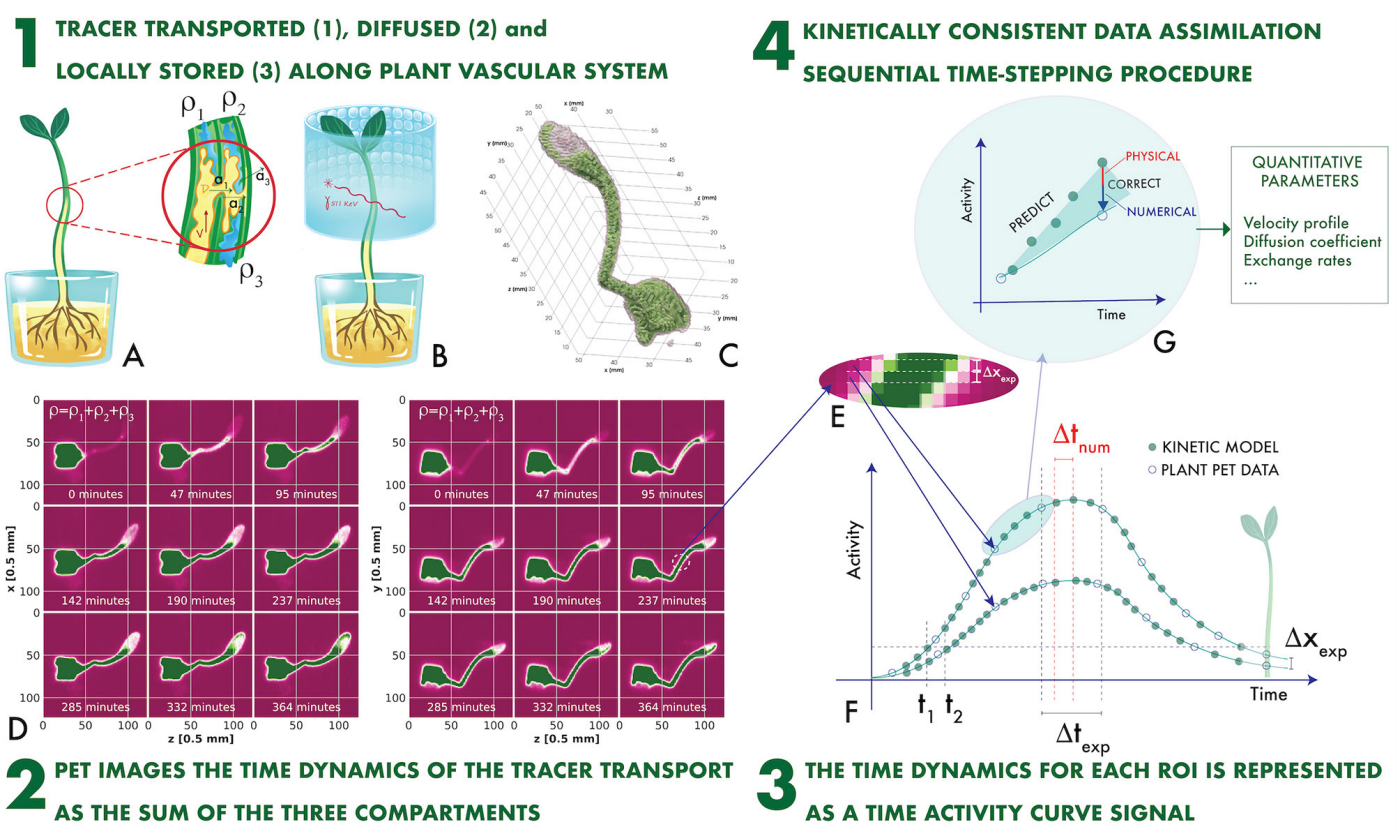
The plant PET imaging technique **(A–F)** and the novel proposed concept of Kinetically Consistent Data Assimilation KC-DA digital signal processing for quantitative plant PET imaging **(F,G)**: the tracer transported, diffused and locally stored along the plant vascular system (1) generates time-changing 3-dimensional maps (2). The change in time of the measured activity at a given region of interest along the stem defines the Time Activity Curve (TAC) signal (3), which is modeled with a time stepping prediction-correction algorithm based on a set of novel fluid dynamic equations for the data-driven extraction of physically-driven kinetic parameters (4).

The plant PET imaging technique is schematically illustrated in Figures 1A–F (Galieni et al., 2021). A ligand, generally H_2_O, CO_2_, or 2-Deoxyglucose (2-DG), is introduced in the plant and is transported in the vascular system (Figure 1A). It is labeled with a β^+^ emitter. Two collinear 511 keV γ rays emerge from the annihilation point of the β^+^ within the plant tissue, are detected in an array of sensors (Figure 1B), and provide tomographic information, which is used to reproduce a time-dependent 3-dimensional map of the ligand displacement in the vascular system (Figures 1C,D).

PET data are extremely sparse in space and time. Intensity maps are sampled with a voxel size Δ*x*_*exp*_ ranging between 0.25 and 1 mm (Kuritaa et al., 2020; Antonecchia et al., 2022). As the minimal spatial resolution of a PET system is approximately 0.54 mm (FWHM) with a ^18^F label in water (Moses, 2011), it is impossible to identify the microscopic capillaries composing the xylem and the phloem. Therefore, voxels are grouped in larger regions of interest (ROI). For instance, a typical ROI structure along the stem of a plant is illustrated in Figure 1E (Mincke et al., 2021a). ROIs with a minimal thickness of one voxel are displaced adjacent to each other. The total activity $\rho \left(x_{i},t^{J}\right)$ measured in the i-th ROI along the stem direction *x* at the time *t*^*J*^ is the TAC signal. It is sampled with a typical time step Δ*t*_*exp*_ of few minutes (Figure 1F).

The time behavior of TAC signals is governed by fluid dynamics (FD) processes (Figure 1A). The measured TAC signal $\rho \left(x_{i},t^{J}\right)$ is the sum of tracer fractions either transported through the xylem $\rho_{1}\left(x_{i},t^{J}\right)$ with a velocity $u\left(x_{i},t^{J}\right)$ and a diffusion coefficient $D\left(x_{i},t^{J}\right)$, or diffused through surrounding parenchyma $\rho_{2}\left(x_{i},t^{J}\right)$ with a rate *a*_1_, or locally assimilated and stored $\rho_{3}\left(x_{i},t^{J}\right)$ with a rate *a*_2_, or diffused through the atmosphere with an efflux rate *a*_3_ (Mincke et al., 2020b).

However, the task of computing the complete fluid state $X_{i}^{J}={\left[\rho_{1},\rho_{2},\rho_{3},u;D,a_{1},a_{2},a_{3}\right]}_{i}^{j}$ from the TAC signal is affected by the sparse nature of the space-time sampling. A simple threshold-based approach illustrates the problem. The apoplastic velocity is responsible of the rising signal front. The average speed *u*_*exp*_ between two adjacent ROIs is *u*_*exp*_ = Δ*x*_*exp*_/(*t*_2_−*t*_1_), where *t*_1_ and *t*_2_ are the times at which the TAC crosses a certain threshold (Figure 1F). The maximal measurable average speed is limited by the sparse sampling, as $u_{exp}^{max}\approx \Delta x_{exp}/\Delta t_{exp}$, with a consequent bias in the determination of the other unknown variables.

To mitigate this intrinsic limitation of TAC signals, data-driven model-free techniques consider only physical parameters with already known validity ranges (Keutgen et al., 2002, 2005; Minchin and Thorpe, 2003; Matsuhashi et al., 2010; Ferrieri et al., 2012) and compartmental modeling estimates only quantities averaged in space and time (Bühler et al., 2011; Hubeau et al., 2018; Mincke et al., 2021a). These approaches cannot be extended to a complete estimation of the FD processes occurring inside the plant vascular system, which remain largely unexplored (Jensen et al., 2012).

Sequential extended Kalman Filter (EKF) data assimilation (DA), based on Bayesian interference, may seem an ideal time-stepping technique for TAC signal processing (Kalman, 1960; Suzuki et al., 2010; Suzuki, 2012; Kato et al., 2015; Suzuki and Yamamoto, 2015; Wang et al., 2017). However, EKF requires a linear implementation of the dynamic model for the prediction of the state ${\tilde{X}}^{J+1}$ at time *J*+1 based on the state *X*^*J*^ at time *J*:


(1) $$\begin{array}{r} {\tilde{X}}^{J+1}=FX^{J} \end{array}$$

Moreover, the physical and numerical errors are modeled with a covariance matrix *P* and an error covariance matrix *Q* (Wang et al., 2017). The predicted covariance matrix ${\tilde{P}}^{J+1}$ at time *J*+1 is evolved from the value *P*^*J*^ at time *J* with the linearized relationship:


(2) $$\begin{array}{r} {\tilde{P}}^{J+1}=FP^{J}F^{T}+Q \end{array}$$

Conventional CFD methods cannot be adapted easily to the form of Equations (1) and (2) as they are based on a finite volume discretization combining central schemes and Riemann solvers for the viscous and inviscid flows, respectively (Issa, 1986; Meldi and Poux, 2017; Qu et al., 2019). Approximated solutions based either on structural similarities between solvers and DA approaches, such as reduced order Kalman filtering (Suzuki, 2012), or on statistical ensemble determination of *P* have been proposed (Evensen, 2009).

In this paper a novel procedure is proposed consisting of assimilating the sparsely sampled TAC signal with computational fluid dynamics (CFD) data simulated at a fine time sampling Δ*t*_*sim*_. A first high resolution predictor stage integrates the FD model forwards in time and, when the experimental data are available, a second correction stage adjusts the model parameters before continuing to the next cycle (Figure 1G). A key aspect of this study is the adoption of a novel set of quasi gas dynamic (QGD) equations for plant PET TAC signals, which can be reduced directly in the form of Equation (1). On this basis a prediction-correction sequential time stepping data assimilation procedure has been developed with the unique feature of a direct computation of the time evolution of the covariance matrix as in Equation (2) without statistical approximations. With respect to existing digital signal processing approaches to plant PET TAC signals, the novel kinetically consistent data assimilation (KC-DA) procedure estimates the complete FD state profile along the vascular system of the plant, controlling explicitly the interplay between physical FD-related mechanisms and computational numerical errors caused by the sparsity of the signal (Figure 1G). It is the first time that Kalman filtering is used in combination with kinetically consistent algorithms for data-driven modeling in plant science. The validity of KC-DA is experimentally demonstrated with an example of zucchini sprouts measurement.

## 2. Materials and Methods

### 2.1. The Kinetically Consistent Data Assimilation Procedure

The transported fluid in the xylem along the 1-dimensional vessel direction *x* was represented by using the time-dependent (*t*) distribution function *f*(**x**, **ξ**, *t*) in the phase-space defined by the local position **x** and velocity **ξ** of the fluid molecules. The macroscopic fluid density and velocity were calculated as the zero-th and first order moments of *f*(**x**, **ξ**, *t*) with respect to the molecular velocity **ξ** (Chapman and Cowling, 1990). The pressure *p*(**x**, *t*) was assumed here to be proportional to the transported tracer density as *p*(**x**, *t*) = *k*_ρ_ρ_1_(**x**, *t*), with *k*_ρ_ proportionality constant. The time evolution of the distribution function was described by the Boltzmann kinetic transport equation (Boltzmann, 1995):


(3) $$\begin{array}{r} \frac{\partial }{\partial t}f\left(\text{x},\xi ,t\right)+\xi_{i}\frac{\partial }{\partial x_{i}}f\left(\text{x},\xi ,t\right)=C\left(f,f^{\prime }\right) \end{array}$$

Equation (3) was solved between equilibrium states, by using a computational time interval Δ*t* proportional to the intrinsic relaxation time τ. Under this approximation, Equation (3) was reformulated in discrete form for the time variable as:


(4) $$\begin{array}{r} \frac{f^{j+1}-f^{j}}{\tau }+\xi_{i}\frac{\partial }{\partial x_{i}}f^{j}=0 \end{array}$$

where the collision integral vanishes, because the transport was effectively computed only at the equilibrium states. The balance equation was obtained by approximating (Equation 4) with a second-order Taylor expansion:


(5) $$\begin{array}{r} \frac{\partial }{\partial t}f\left(\text{x},\xi ,t\right)+\xi_{i}\frac{\partial }{\partial x_{i}}f\left(\text{x},\xi ,t\right)=\\ \;=\frac{\tau }{2}\xi_{i}\xi_{k}\frac{\partial }{\partial x_{i}}\frac{\partial }{\partial x_{k}}f\left(\text{x},\xi ,t\right) \end{array}$$

The zero-th order momentum of Equation (5) expressed an equation for ρ_1_(*x, t*) (Chetverushkin, 2015; Chetverushkin et al., 2017), which was coupled with the dynamic equations regulating the contributions of ρ_2_(*x, t*) and ρ_3_(*x, t*) (Mincke et al., 2021a), defining the system of QGD equations for plant PET TAC signals:


(6) $$\begin{array}{r} \frac{\partial }{\partial t}\rho_{1}\left(x,t\right)+\frac{\partial }{\partial x}\rho_{1}\left(x,t\right)u\left(x\right)=D\frac{\partial }{\partial x^{2}}\rho_{1}\left(x,t\right)+\\ +\frac{\tau \left(x\right)}{2}\frac{\partial }{\partial x^{2}}\left[\rho_{1}\left(x,t\right)u^{2}\left(x\right)\right]-a_{1}\rho_{1}\left(x,t\right) \end{array}$$

(7) $$\begin{array}{r} \frac{\partial }{\partial t}\rho_{2}\left(x,t\right)=a_{1}\rho_{1}\left(x,t\right)-a_{2}\rho_{2}\left(x,t\right)-a_{3}\rho_{2}\left(x,t\right) \end{array}$$

(8) $$\begin{array}{r} \frac{\partial }{\partial t}\rho_{3}\left(x,t\right)=a_{2}\rho_{2}\left(x,t\right) \end{array}$$

The terms in the left side of Equation (6) defines the apoplastic flow. The first term in the right side of Equation (6) represents the transcellular roots, with a macroscopic diffusion coefficient D. A remarkable feature of this model is, that the second but last term of Equation (6) introduces explicitly the numerical viscosity caused by the sparse sampling Δ*x*_*exp*_ of the TAC signal, with τ(*x*) = α_τ_Δ*x*_*exp*_/*u*(*x*), where α_τ_ is a tunable parameter. The coefficients *a*_*i*_ in Equations (7) and (8) represent the exchange rates between the different processes. Temperature, humidity and illumination are usually controlled in plant imaging experiments. The model was restricted to observations performed within few hours and along segments of the stem, which never exceded approximately 10 cm. Therefore, the velocity *u*(*x*) was considered constant in time and the diffusion coefficient D constant both in space and time. By using a time-explicit and central spatial numerical discretization scheme, with N spatial steps of variable size Δ*x*_*exp, i*_ and time steps of variable length $\Delta t_{sim}^{j}$, the system of Equations (6–8) was expressed in the form of Equation (1), with *F* a (4*N*+4) × (4*N*+4) array defined in Table 1 and border conditions ρ_*i*, 0_ = ρ_*i*, 1_, ρ_*i, N*−1_ = ρ_*i, N*_, *u*_*i*, 0_ = *u*_*i*, 1_, *u*_*i, N*−1_ = *u*_*i, N*_. While the TAC spatial sampling Δ*x*_*exp, i*_ is not interpolated, $\Delta t_{sim}^{j}$ is generally smaller than the TAC time sampling $\Delta t_{exp}^{J}$. Therefore, $\Delta t_{exp}^{J}$ was decomposed in a series of finer $\Delta t_{sim}^{j}$. The indices *J* and *j* refer to experimental and simulated time sampling, respectively.

**Table 1:** The evolution matrix *F*.

***F*_*ik*_≠0**	***i*_*min*_**	***i*_*max*_**	**k**
$\frac{\Delta t_{sim}^{j}}{2\Delta x_{exp,i}}u_{i-1}^{j}+\frac{\tau u_{i-1}^{j2}\Delta t_{sim}^{j}}{\Delta x_{exp,i}^{2}}+$			
$+D\frac{\Delta t_{sim}^{j}}{\Delta x_{exp,i}^{2}}$	1	*N*−2	*i*−1
$1-\frac{2u_{i}^{j2}\tau \Delta t_{sim}^{j}}{\Delta x_{exp,i}^{2}}-2\frac{D\Delta t_{sim}^{j}}{\Delta x_{exp,i}^{2}}-$			
$a_{1}\Delta t_{sim}^{j}$	1	*N*−2	*i*
$-\frac{\Delta t_{sim}^{j}}{2\Delta x_{exp,i}}u_{i+1}^{j}+\frac{\tau u_{i+1}^{j2}\Delta t_{sim}^{j}}{\Delta x_{exp,i}^{2}}+$			
$D\frac{\Delta t_{sim}^{j}}{\Delta x_{exp,i}^{2}}$	1	*N*−2	*i*+1
$a_{1}\Delta t_{sim}^{j}$	*N*+1	2*N*−2	*i*−*N*
$-a_{2}\Delta t_{sim}^{j}-a_{3}\Delta t_{sim}^{j}$	*N*+1	2*N*−2	*i*
$a_{2}\Delta t_{sim}^{j}$	2*N*+1	3*N*−2	*i*−*N*
1	3*N*	4*N*+3	*i*

*The elements with indices i and k outside the indicated bounds vanish*.

The complete FD state was a (4*N*+4)-dimensional array:


(9) $$\begin{array}{l} XJ^{}=[\rho_{1,0},\ldots ,\rho_{1,N},\rho_{2,0},\ldots ,\rho_{2,N},\rho_{3,0},\ldots ,\rho_{3,N},u_{0},\\ \;{\ldots ,u_{N};D,a_{1},a_{2},a_{3}]}^{J} \end{array}$$

The TAC signal at the time *J* and at the ROI *i* was expressed as the sum of the transported, diffused and assimilated tracer fractions $\rho_{i}^{J}=H_{i,k}X_{k}{}^{J}$, with *H*_*i, k*_ = δ_*i, k*_+δ_*i*+*N, k*_+δ_*i*+2*N, k*_.

The uncorrelated uncertainties related to the experimental measurement $\sigma_{\rho }^{exp}$ was modeled as the diagonal *N*×*N* matrix $R_{i,k}=\sigma_{\rho }^{exp}\delta_{i,k}$. The theoretical uncertainty of the estimation of the density $\sigma_{\rho }^{th}$ affects the error of the estimated velocity, diffusion coefficient and exchange rates. Following this assumption, we approximated the time-dependent (4*N*+4) × (4*N*+4) process noise variance as *Q* = *VTV*^*T*^, where $T_{i,k}=\sigma_{\rho }^{th}\delta_{i,k}$ and *V* is the (4*N*+4) × (*N*+4)-dimensional Jacobian defined in Table 2.

**Table 2:**
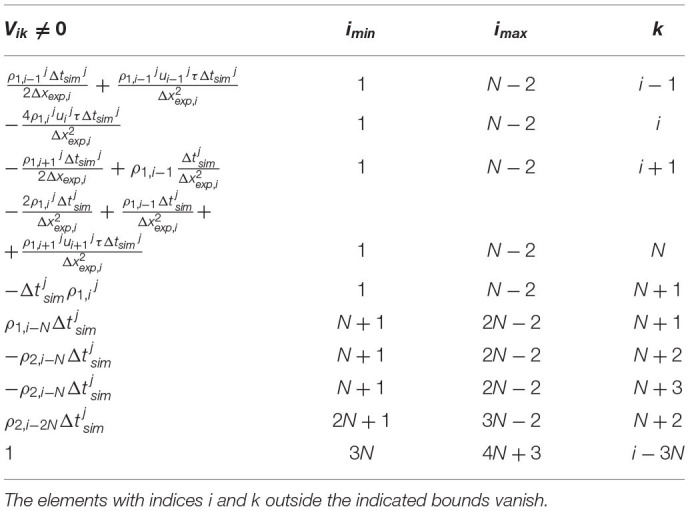
The Jacobian matrix *V*.

*The elements with indices i and k outside the indicated bounds vanish*.

The novel KC-DA procedure was based on the minimization of the time-dependent covariance of the physical system *P_i,k_^J^*, which was initialized as a diagonal matrix, with an initial guess of the theoretical errors of the state variables. The initial value of $\rho_{1}^{0}$ was defined from the corresponding measurement ρ^0^, while $\rho_{2}^{0}$ and $\rho_{3}^{0}$ vanished. The KC-DA procedure, as shown in Table 3, resulted in a predictor-corrector approach. A predictor step estimates the expected value of the TAC and a corrector step adapts the prediction to the measurement:

**a. Predictor step** The predicted values of the state variables $\tilde{X}$ and of the covariance matrix $\tilde{P}$ are calculated with Equations (1) and (2). $\Delta t^{j}{}_{sim}$ is adjusted adaptively as $\Delta t^{j}{}_{sim}=\alpha \times \min\left(\Delta x_{\exp,i}/u_{i}{}^{j}\right)$, where α is the Courant parameter.

**b. Corrector step** If data are available at the time *J*+1, then *X* and *P* are updated as Wang et al. (2017):


(10) $$\begin{array}{r} X^{J+1}={\tilde{X}}^{J+1}+K\left(\rho^{J+1}-H{\tilde{X}}^{J+1}\right)\\ P^{J+1}={\tilde{P}}^{J+1}\left[I-KH\right]\\ K=\left[{\tilde{P}}^{J+1}H^{T}{\left(H{\tilde{P}}^{J+1}H^{T}+R\right)}^{-1}\right] \end{array}$$

**Table 3:**
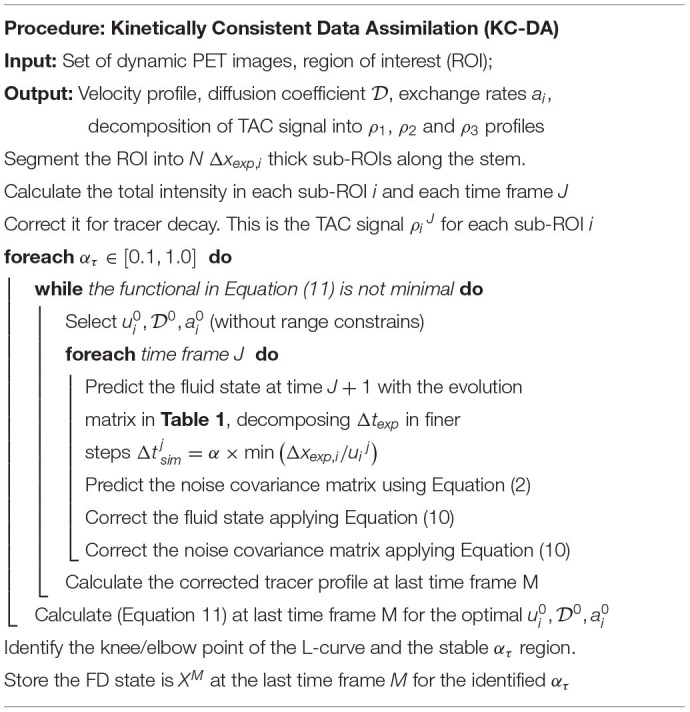
A schematic description of the implementation of the KC-DA procedure.

The value of *u*_*i*_, D and *a*_*i*_ were obtained from the state vector *X*^*j*^ at the last time step *M*. Initial settings may cause a bias in the convergence of the filter. Therefore, an optimization discrepancy functional for $u_{i}^{0},D^{0},a_{i}^{0}$ has been added:


(11) $$\begin{array}{r} \phi \left(u_{i}^{0},D^{0},a_{i}^{0}\right)=\overset{N}{\underset{i=0}{\sum }}\left[{\left(\rho -HX^{M}\right)}_{i}^{2}+{\left(\rho -H{\tilde{X}}^{M}\right)}_{i}^{2}\right] \end{array}$$

A sequential least squares programming minimization algorithm for Equation (11) in the KC-DA procedure was used.

### 2.2. Simulated Data

The validation of the KC-DA method was first performed by using simulated data. A realistic spatial profile of ρ_1_ extracted from an existing dataset was set as initial condition, with ρ_2_ and ρ_3_ initially vanishing. The parameters of the model variated within given ranges. The velocity profile was set constant in the range *v*_0_∈(0.05, 0.15). The other fluid dynamic parameters were set in the ranges *D*_0_∈(0.043, 0.093), *a*_0_∈(0.0031, 0.0081), *a*_1_∈(0.0026, 0.0076). The time evolution of the profile was simulated by using the predictor function in Equation (1) at equally spaced (15 min) time steps in the range (0, 340) min. The KC-DA algorithm was applied to estimate the fluid dynamic parameters from the simulated profiles and the difference between the estimated and the true parameters was measured.

### 2.3. Plant Experiments

The KC-DA algorithm was tested on PET TAC signals of sprouts of zucchini (*Cucurbita pepo L., var. Genovese*, Four Sementi, Piacenza, Italy). A group of 20 plants was selected, grown in the same controlled environment, 10 days after sowing. The roots of all plants were immersed in a 80 μCi solution of 2-[^18^F]-FDG diluted in 1 cc water and a 340 min long dynamic scan (RAYCAN E180;Liang et al., 2020) was performed with Δ*t*_*exp*_ = 15 min. The length of the stem, weight and final activity were on average 6 cm, 0.72 g and 2 μCi, respectively. A 35 mm long ROI with Δ*x*_*exp*_ = 0.5 mm was selected. α = 0.4 and an initial 5% estimate of the experimental, measurement and model errors were set. The rate *a*_3_ was assumed to vanish, as it refers to gaseous tracers transpiration (Mincke et al., 2020b).

## 3. Results

### 3.1. Numerical Verification of KC-DA

**Figure 2:**
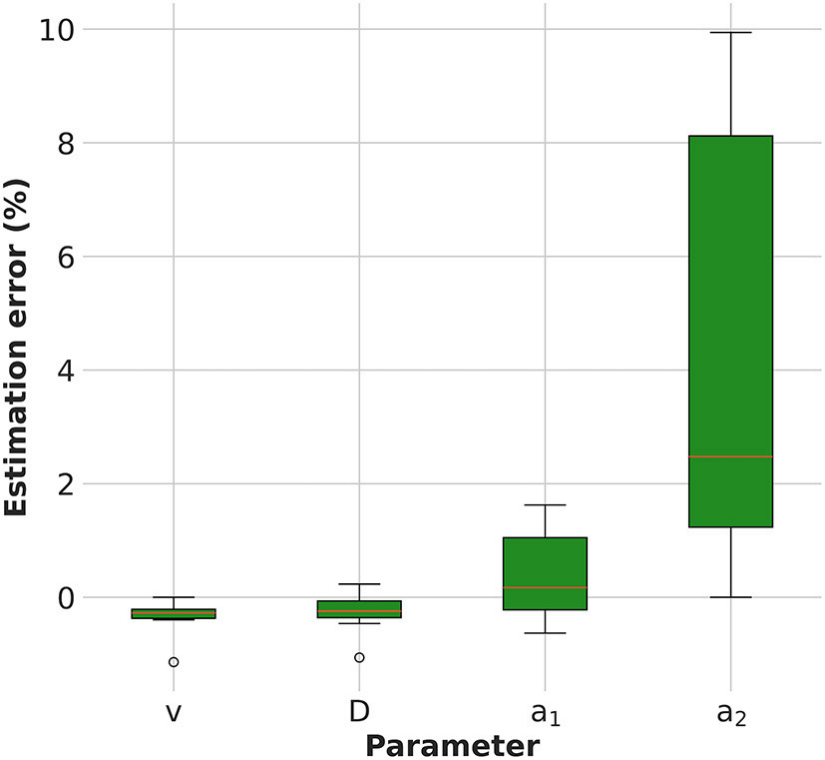
Numerical verification of the KC-DA algorithm: estimation error of the model parameters.

The estimation error of the model parameters is shown in Figure 2. The KC-DA algorithm applied to simulated data was able to retrieve the model parameters with a relative error on average lower than 5% (FWHM) and with an average relative bias of –0.1, –0.1, 0.2, and 2.3% for the velocity, viscosity coefficient D and exchange rates *a*_1_ and *a*_2_, respectively.

### 3.2. Convergence and Physical Significance of the KC-DA

**Figure 3:**
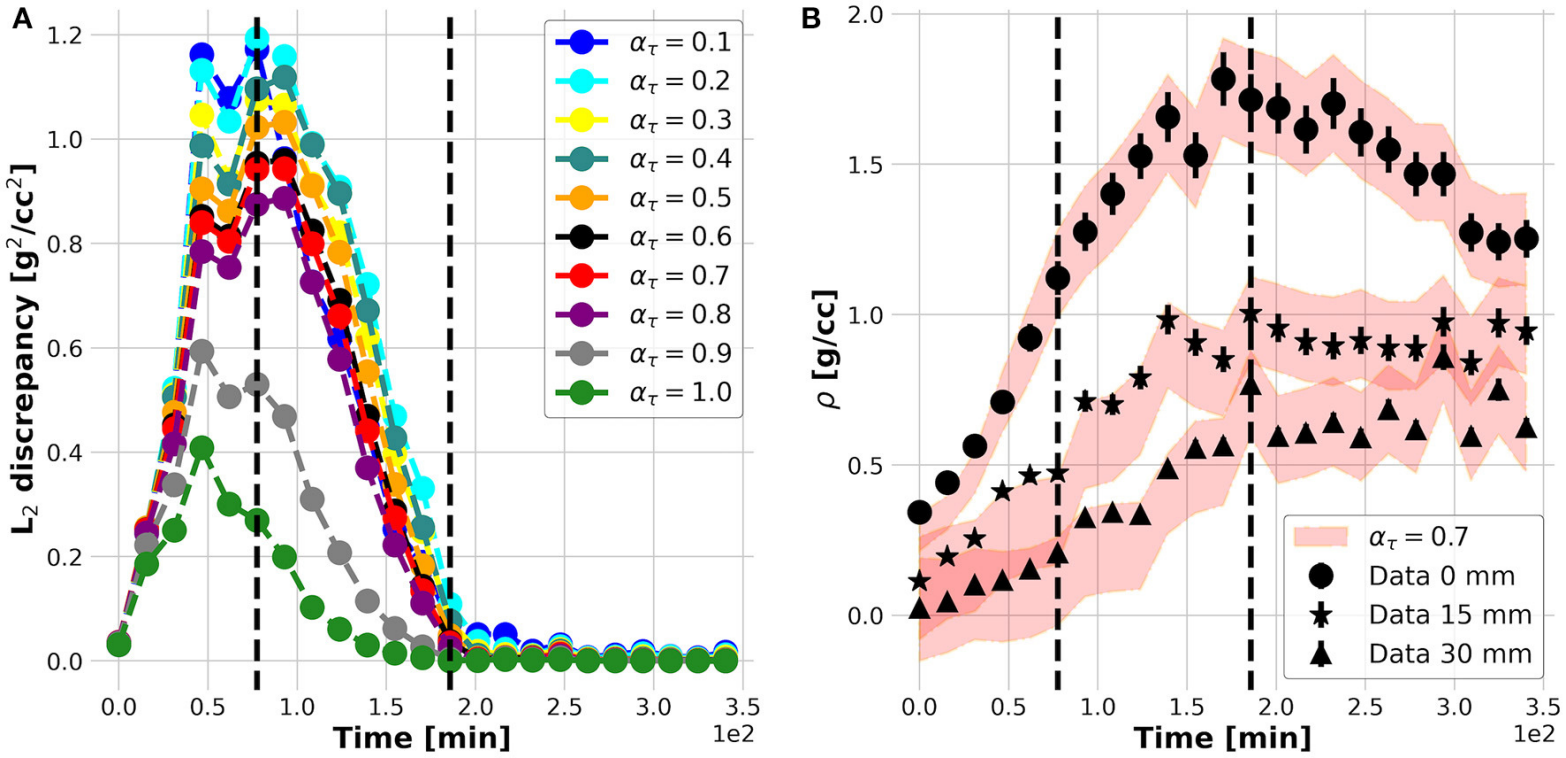
L_2_ discrepancy between model prediction (filled red band) and data (markers) for different α_τ_ **(A)**; measured (filled dots) and predicted (red bands) time profiles at three equally spaced positions along the plant stem **(B)**.

The results of the plant experiments are further reported following and clarifying each logic step of the nested structure of the KC-DA procedure summarized in Table 3. The internal **foreach**-loop represents the sequential time-stepping data assimilation approach to parameter estimation. As shown in Figure 3A, after an initial increase, the L_2_ discrepancy between model prediction and data reaches a maximal value at *t* = 70 min and decreases further until *t* = 200 min, indicating an increasing match between the estimated parametric set up and the TAC signal. The action of the data-driven learning mechanism occurring in the internal **foreach**-loop is visible in Figure 3B, where the TAC signal at three equally spaced ROIs is shown. The model prediction matched the TAC signal with increasing precision after *t* = 70 min.

**Figure 4:**
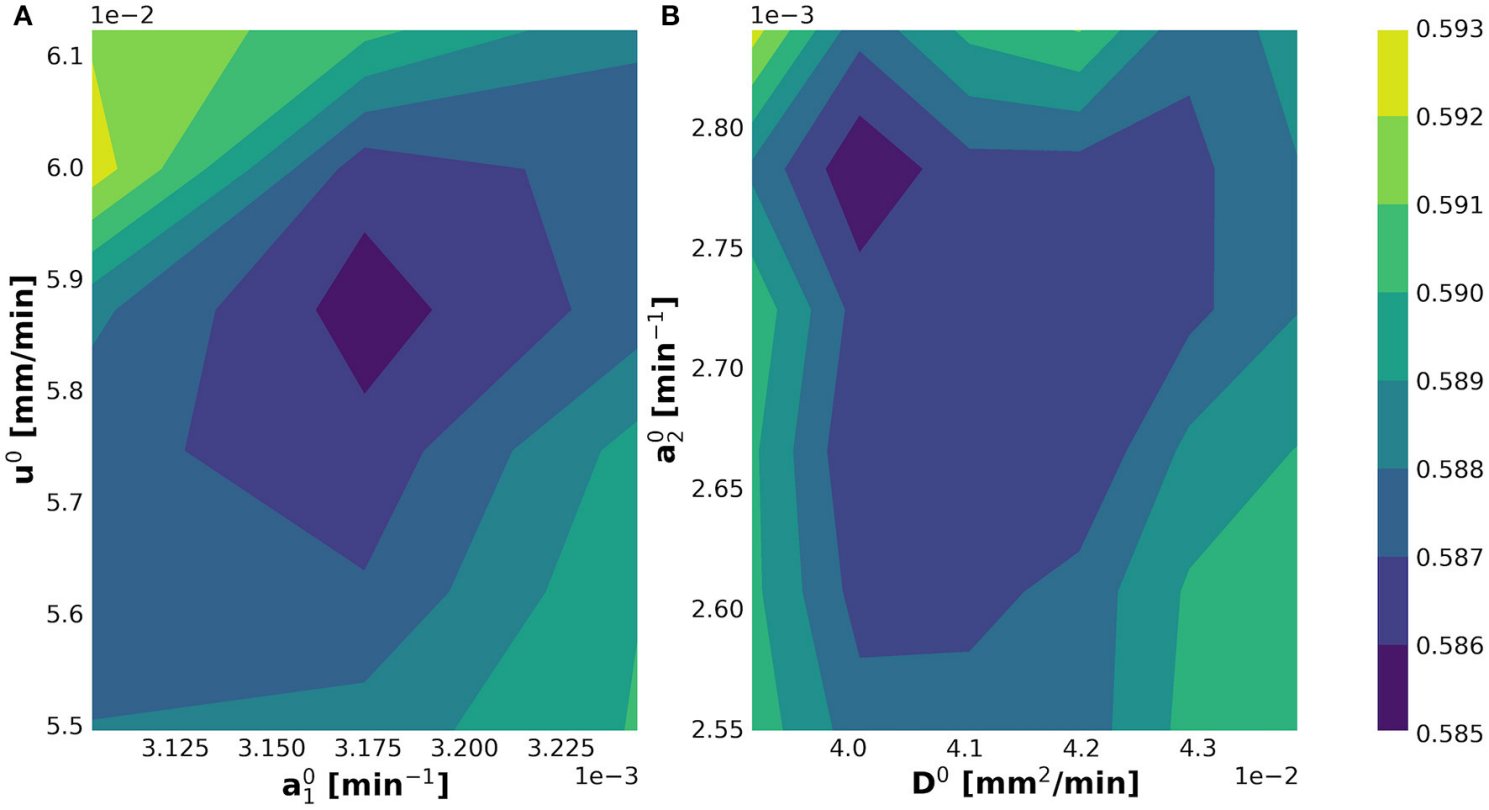
Determination of the minimum of the optimization discrepancy functional in Equation (11) shown in the $u^{0}-a_{1}^{0}$ **(A)** and $a_{2}^{0}-D^{0}$ **(B)** planes.

The internal **while**-loop searches for the optimal parametric set up for the initialization of the KC-DA procedure. The 2-dimensional profiles of the discrepancy functional in Equation (11) verified that the algorithm converged to a well-identified minimum for the initial set of parameters (Figures 4A,B).

**Figure 5:**
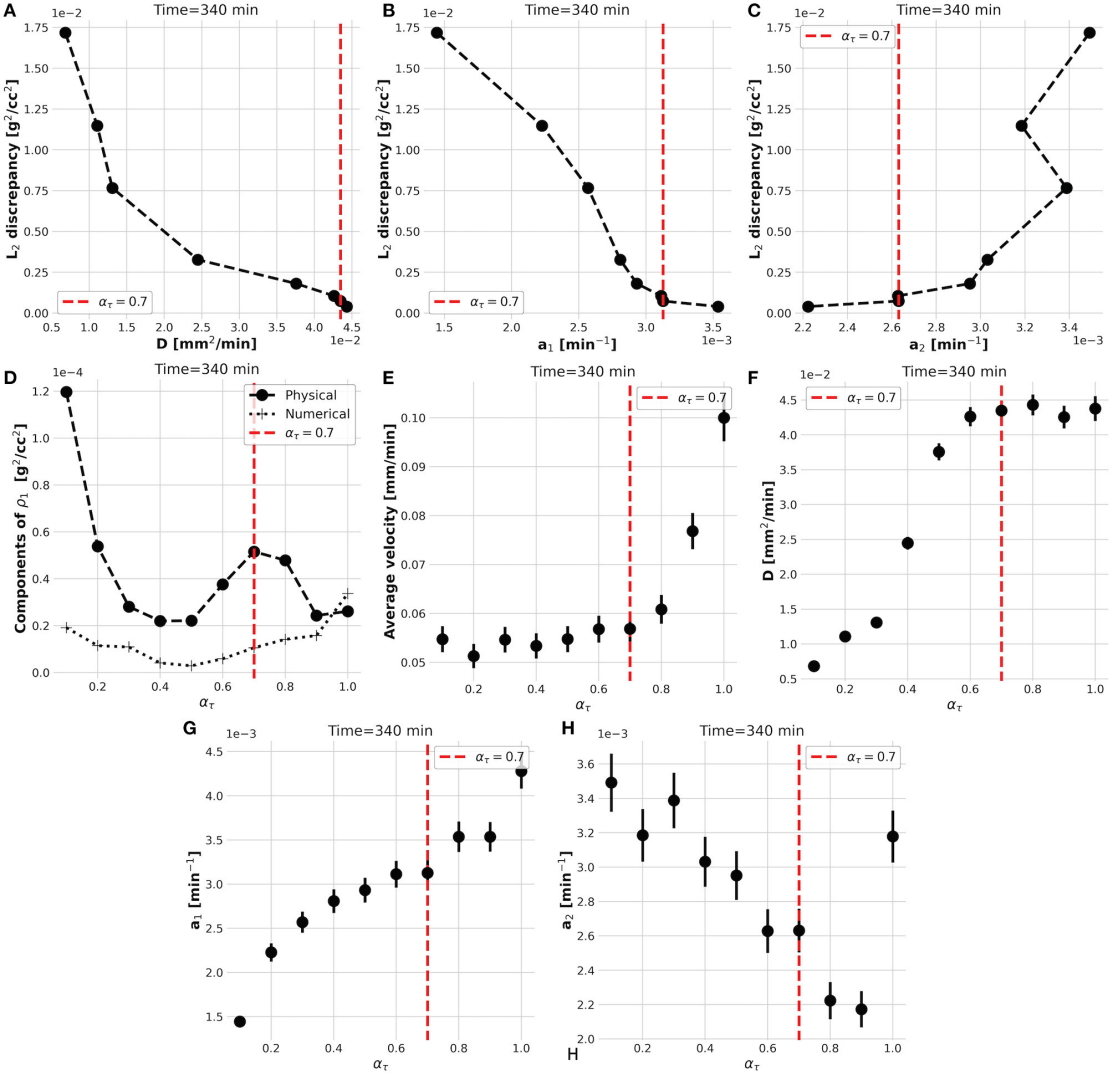
The L-shaped functional dependence between L_2_ discrepancy and estimated parameters at different α_τ_ **(A–C)**, impacts the strength of the physical and numerical dissipative components of the KC-DA procedure **(D)**. The knee/elbow point of the *L*_2_ discrepancy at α_τ_ = 0.7 (red dotted line) identifies the best estimation of the parameters, which corresponds to a plateau region **(E–H)**.

However, the convergence of the **while**-loop is guaranteed for any α_τ_. Therefore, the scope of the external **foreach**-loop of the KC-DA procedure is to identify the physically-significant choice of α_τ_. The L_2_ discrepancy between model prediction and data decreases on average with α_τ_ (Figure 3A). This trend generated the typical L-shaped relationship between L_2_ discrepancy and estimated parameters (Figures 5A–C). A unique feature of the KC-DA procedure is the explicit calculation of the physical and numerical viscosity components, which accounts for the effect of the physical diffusion and of the spatial sparsity of the TAC signal, respectively. The dependence of these two components over α_τ_ is particularly explicative of the physical mechanism of the external **foreach**-loop of the KC-DA procedure. As visible in Figure 5D, the progressive descent of the D and a_1_ L-curves for α_τ_ ≤ 0.5 (Figures 5A,B) corresponded to the decreasing strength of the physical dissipation. At approximately α_τ_ = 0.7, all the L-curves stabilize after the knee/elbow point and the physical dissipation increased again reaching a maximal value. For α_τ_≥0.7 the L-curves had a very slow decrease, but the physical dissipation diminished abruptly and became illogically comparable to the numerical dissipation. The L-curve for a_2_ followed an opposite trend with respect to a_1_ and D, but confirmed the stability after the knee/elbow point (Figure 5C). The value of the predicted fluid parameters exhibited also a dependence on α_τ_, reaching a short plateau at α_τ_≈0.6−0.7 (Figures 5E–H). This stable region after the knee/elbow point is the proper choice for α_τ_.

**Figure 6:**
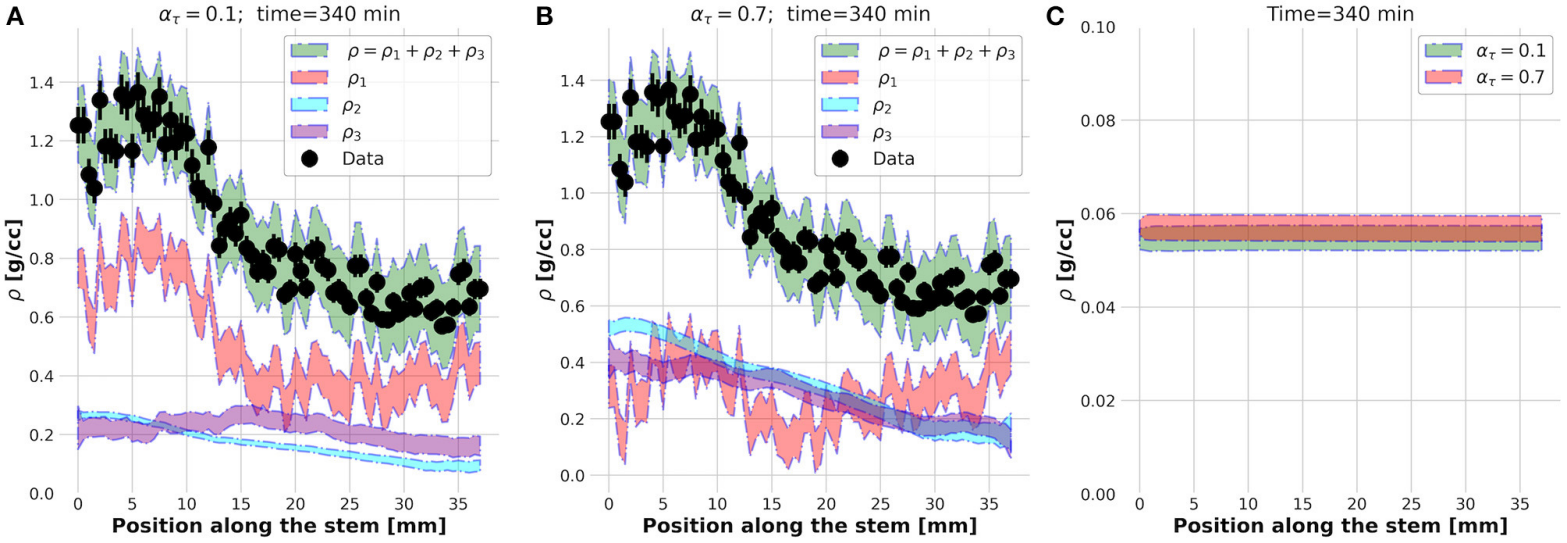
Estimated spatial profiles of the ρ_1_, ρ_2_, and ρ_3_ components of the tracer density ρ at time *t* = 340 s for α_τ_ = 0.1 **(A)** and α_τ_ = 0.7 **(B)**. Velocity profile at *t* = 340 s **(C)**.

The physical meaning of this external **foreach**-loop of the KC-DA procedure is better explained looking at the spatial profiles of ρ_1_, ρ_2_, ρ_3_, and ρ estimated at α_τ_ = 0.1 (Figure 6A) and α_τ_ = 0.7 (Figure 6B). Although in both cases the predicted ρ was found in excellent agreement with the data, at α_τ_ = 0.1 the xylem transport component ρ_1_ was dominant, while at α_τ_ = 0.7 the parenchyma diffusion ρ_2_ and the local assimilation ρ_3_ played a major contribution. As the FD state was predicted at *t* = 340 min, it was reasonable to expect that most of the transport flow already almost vanished and the fluid diffusion in the apoplastic spaces and subsequent local storage were the dominant processes, therefore supporting the findings at α_τ_ = 0.7. The apoplastic velocity profile of the transported ρ_1_ in the two cases was almost equivalent (Figure 6C). It could be concluded that the region right after the knee/elbow point of the L-curve computed in the external **foreach**-loop identified the data assimilation model which learned and predicted the correct physical behavior of the plant dynamic flow.

The estimated parameters were D=(0.043±0.001)mm^2^/min, *a*_1_ = (0.0031±0.0002)min^−1^, *a*_2_ = (0.0026±0.0001)min^−1^, and < *u*>(0.058±0.02)mm/min, where the errors include the variation (FWHM) across the entire dataset.

## 4. Discussion

The findings presented above approach the problem of a quantitative interpretation of the dynamic plant PET data from a physical and computational point of view. The key advance of KC-DA with respect to the state of the art is the possibility of the extraction of a continuous profile of the FD variables along the plant stem based on a realistic dynamic physical model, as shown in Figure 6. The relevance of novel mathematical modeling based on biophysical mechanisms has been recently emphasized (Tredenick and Farquhar, 2021) and models which describe the mechanistic properties of water movement in the different parts of plants became fundamental for the interpretation of measurements using nuclear science approaches, such as PET. Although these models are based on fluid dynamics and are extended with compartmental modeling for exchange rates between different functional compartments in plant tissues (Bühler et al., 2011; Mincke et al., 2021b), they are able to extract only an averaged quantities along small sections of the stem. Similarly, model-free techniques such as input-output approaches (Matsuhashi et al., 2010) can calculate only the mean speed of tracer transport and the proportion of tracer moved between specified image positions by means of transfer function analysis. For instance, it has been observed with these techniques that local variations of the average transport speed of water between 0.7 and 1.8 cm/min occurs at different parts of the stem of *Sorghum* (Keutgen et al., 2005). It is interesting to note that, from the difference of tracer arrival times at three equally spaced points along the stem of a soybean plant, it has been estimated that water transport occurs with an approximate constant speed of 4 mm/s in a total length of 30 mm (Ohya et al., 2008). These two apparently contradictory results are well representative of the need of a precise estimation of the continuous profiles along longer segments of the stem, as proposed in this paper (Figure 6), which will support agronomists in the estimation of such interesting and still unexplored quantitative feature of the plant, which are otherwise not directly accessible with other experimental techniques.

From a mathematical point of view, KC-DA approaches the problem of a reliable estimation of the correlation matrix between the parameters. While typical approaches include either a sensitivity analysis or a Monte Carlo based error estimation (Bühler et al., 2011; Mincke et al., 2021b), KC-DA has the distinctive feature of a direct computation of the correlation matrix *P* as in Equation (2), which takes also into account the numerical errors due to the discretization of the computational mesh used in the predictor and to the intrinsic voxel size of the measured PET data (Figure 5D). The results in Figure 2 show that the method exhibits a good numerical stability and precision.

A limit of KC-DA is that the convergence improves with time as shown in Figure 3A, and the initial frames of the tracer dynamic cannot be estimated with precision. This feature has a direct effect on the estimated parameters. While the viscosity coefficient and the exchange rates are found in an expected range (Bühler et al., 2011; Mincke et al., 2020b), the velocity profile appears almost constant and with an average value low in comparison with the above mentioned results. As observed in Figures 6A,B, this occurs as, while apoplastic flow dominates at the initial stages of the tracer immersion, at later times the diffusive flow is dominant. Such effect suggests that the dynamic model in Equation (8) could be extended by including a time-dependent velocity, allowing therefore to precisely capture the tracer dynamics at early stages. The next step of this study will be to demonstrate the validity of the KC-DA approach with more conservation equations and ligands used in plant science, toward an increasingly complex modeling of plant PET TAC signals.

## 5. Conclusions

A key feature of KC-DA is the ability of calculating the continuous profile of kinetic variables associated to the FD flow in plant transport. This makes KC-DA particularly suited to the quantification of plant vascular flow. KC-DA has an explicit computational implementation thanks to the analytical form of the evolution and noise correlation matrices. The direct calculation of the numerical dissipative terms plays a pivotal role in the stabilization of the time stepping procedure and helps the stability of the estimation of the transport parameters in the plant vascular system.

## Data Availability Statement

The raw data supporting the conclusions of this article will be made available by the authors, without undue reservation.

## Author Contributions

ND'A ideated the data-driven kinetically consistent approach and wrote the analysis code. ND'A and MP analyzed the data and wrote the manuscript. EA, MC, GP, and MP designed the possible experiments and applications to agronomy. ND'A, MP, and QX supervised the research focus and designed the scientific background. All authors contributed to the article and approved the submitted version.

## Conflict of Interest

The authors declare that the research was conducted in the absence of any commercial or financial relationships that could be construed as a potential conflict of interest.

## Publisher's Note

All claims expressed in this article are solely those of the authors and do not necessarily represent those of their affiliated organizations, or those of the publisher, the editors and the reviewers. Any product that may be evaluated in this article, or claim that may be made by its manufacturer, is not guaranteed or endorsed by the publisher.
